# Association between Estimated Pulse Wave Velocity (ePWV) and in-hospital and ICU 28-day mortality in ischemic stroke patients: A retrospective analysis of the MIMIC-IV database

**DOI:** 10.1371/journal.pone.0328818

**Published:** 2025-08-12

**Authors:** Shuhe Zhao, Mingjie Liu, Minheng Zhang, Hongwei Liu, Xuan Chen, Yu Wang

**Affiliations:** 1 Department of Neurology, Taiyuan City Central Hospital, The Ninth Clinical Medical College of Shanxi Medical University, Taiyuan, Shanxi Province, China; 2 Department of Oncology, Liyuan Hospital, Tongji Medical College, Huazhong University of Science and Technology, Wuhan, Hubei Province, China; 3 Department of Gerontology, The First People’s Hospital of Jinzhong, Yuci, Shanxi Province, China; 4 Department of Emergency Internal Medicine, Shanxi Bethune Hospital, Shanxi Academy of Medical Sciences, Third Hospital of Shanxi Medical University, Tongji Shanxi Hospital, Taiyuan, Shanxi Province, China; Royal Holloway University of London, UNITED KINGDOM OF GREAT BRITAIN AND NORTHERN IRELAND

## Abstract

**Background:**

Ischemic stroke poses a substantial global health burden. Reliable biomarkers for risk stratification in critically ill stroke patients are lacking. This study investigates estimated pulse wave velocity (ePWV), a non-invasive measure of arterial stiffness, as a novel prognostic indicator for mortality in this population.

**Methods:**

This retrospective cohort study analyzed data from 3,408 adult ischemic stroke patients admitted to the ICU within the MIMIC-IV database. Patients were categorized by ePWV tertiles. The primary outcome was 28-day mortality (in-hospital and ICU). Multivariate Cox regression models were employed to assess the association between ePWV and mortality, adjusting for comprehensive clinical variables.

**Results:**

Of the 3,408 patients, 481 (14.1%) died within 28 days of hospitalization. Non-survivors demonstrated significantly higher ePWV values (11.19 vs. 10.57, *P* < 0.001). Multivariate analysis revealed that ePWV was an independent predictor of both in-hospital (HR = 1.16, 95% CI: 1.05–1.28, *P* = 0.0033) and ICU 28-day mortality (HR = 1.31, 95% CI: 1.16–1.48, *P* < 0.0001). Subgroup analyses revealed significant interactions between ePWV and atrial fibrillation for in-hospital mortality (*P* = 0.0498) and mechanical ventilation for ICU mortality (*P* = 0.0294). For in-hospital mortality, the ePWV-associated risk was higher in patients with atrial fibrillation (HR 1.19, 95% CI: 1.07–1.31) compared to those without (HR 1.10, 95% CI: 0.98–1.23). For ICU mortality, the ePWV-associated risk was higher in patients without mechanical ventilation (HR 1.45, 95% CI: 1.24–1.70) compared to those with (HR 1.26, 95% CI: 1.11–1.44).

**Conclusion:**

ePWV is a promising biomarker for predicting mortality in critically ill ischemic stroke patients, particularly identifying high-risk subgroups with atrial fibrillation or those not receiving timely mechanical ventilation.

## Introduction

Ischemic stroke, characterized by the interruption of blood flow to the brain, is a leading cause of morbidity and mortality worldwide [1]. The pathophysiology of ischemic stroke involves a complex interplay of vascular occlusion, inflammation, and neuronal injury, which can lead to rapid brain tissue death if not promptly addressed [2]. The burden of ischemic stroke is compounded by its association with various risk factors, including hypertension, diabetes, and hyperlipidemia, which contribute to the underlying vascular pathology [3]. Given the high incidence and the profound impact of ischemic stroke on patients and healthcare systems, understanding its prognostic factors is crucial for improving clinical outcomes.

Previous studies have highlighted the utility of various clinical scoring systems and biomarkers in predicting outcomes in critically ill patients, including those with ischemic stroke [4,5]. For instance, the Sequential Organ Failure Assessment (SOFA) and Simplified Acute Physiology Score II (SAPS II) scores are commonly utilized to evaluate the severity of illness and predict mortality in critically ill patients [6,7]. However, there remains a need for more reliable and easily applicable prognostic tools that can enhance risk stratification in this vulnerable population. In contrast to these conventional scores that primarily evaluate organ dysfunction (e.g., SOFA) or acute physiological status (e.g., SAPS II), estimated pulse wave velocity (ePWV) enables more nuanced characterization of cardiovascular status, potentially refining risk stratification. The ePWV is a non-invasive measure of arterial stiffness, which is calculated using age and mean blood pressure (MBP) [8]. This estimation is based on established equations derived from the Reference Values for Arterial Stiffness Collaboration, which correlate well with the gold standard measurement of carotid-femoral pulse wave velocity (cfPWV) [9]. The calculation of ePWV allows for a practical assessment of vascular health in clinical settings, particularly in populations where direct measurement of cfPWV may not be feasible. The ePWV is increasingly recognized as a significant marker of cardiovascular health, reflecting the elasticity of arterial walls. Elevated ePWV values are associated with various cardiovascular risk factors, including hypertension, diabetes, and aging [10]. Studies have demonstrated that increased arterial stiffness, as indicated by higher ePWV, correlates with adverse cardiovascular outcomes, including myocardial infarction and stroke [11]. Furthermore, ePWV has been shown to predict cardiovascular mortality independently of traditional risk factors, highlighting its potential role in risk stratification and management of patients with cardiovascular diseases [12]. The study by Stamatelopoulos et al. revealed that incorporating ePWV enhances the prognostic value of existing clinical scoring methods through its unique capacity to provide vascular health insights [13]. Their findings demonstrated that ePWV provided significant discrimination enhancement and reclassification advantages over the 4C Mortality score and Charlson comorbidity index (CCI).

The relationship between ePWV and cardiovascular health underscores the importance of monitoring arterial stiffness as part of comprehensive cardiovascular risk assessment [14]. However, a critical gap remains in our understanding of ePWV’s association with short-term mortality among critically ill ischemic stroke patients, particularly within the ICU setting. Previous research on ePWV in stroke has primarily focused on long-term outcomes in non-institutionalized stroke populations [15,16], largely neglecting the unique challenges and complexities presented by critically ill patients. Moreover, while studies on critically ill patients have explored ePWV in conditions such as acute kidney injury, subarachnoid hemorrhage, and coronary heart disease [17–19], this specific population of critically ill ischemic stroke patients has not been directly examined. Therefore, this study aims to evaluate the predictive value of ePWV for in-hospital and ICU 28-day mortality in patients with ischemic stroke using data from the MIMIC-IV database. We hypothesize that elevated ePWV will be independently associated with increased in-hospital and ICU 28-day mortality in this population. By conducting a retrospective analysis, we seek to determine whether ePWV can serve as an independent predictor of mortality in this population, thereby enhancing risk stratification and informing clinical management strategies.

## Methods

### Study design and population

The analysis utilized data extracted from the Medical Information Mart for Intensive Care (MIMIC-IV version 3.0) database, which contains comprehensive clinical information on critically ill patients [20,21]. The author of this study, Mingjie Liu (ID: 13908736), registered, completed necessary training, and obtained approval to use the dataset for this study. The database contains comprehensive medical records of patients admitted to the intensive care units at Beth Israel Deaconess Medical Center, which provides a rich resource for analyzing patient outcomes in various critical care settings [22]. The use of the MIMIC-IV database was approved by the Institutional Review Boards of the Massachusetts Institute of Technology (MIT) and Beth Israel Deaconess Medical Center (BIDMC). Since the data were anonymized, informed consent was not required.

**Fig 1:**
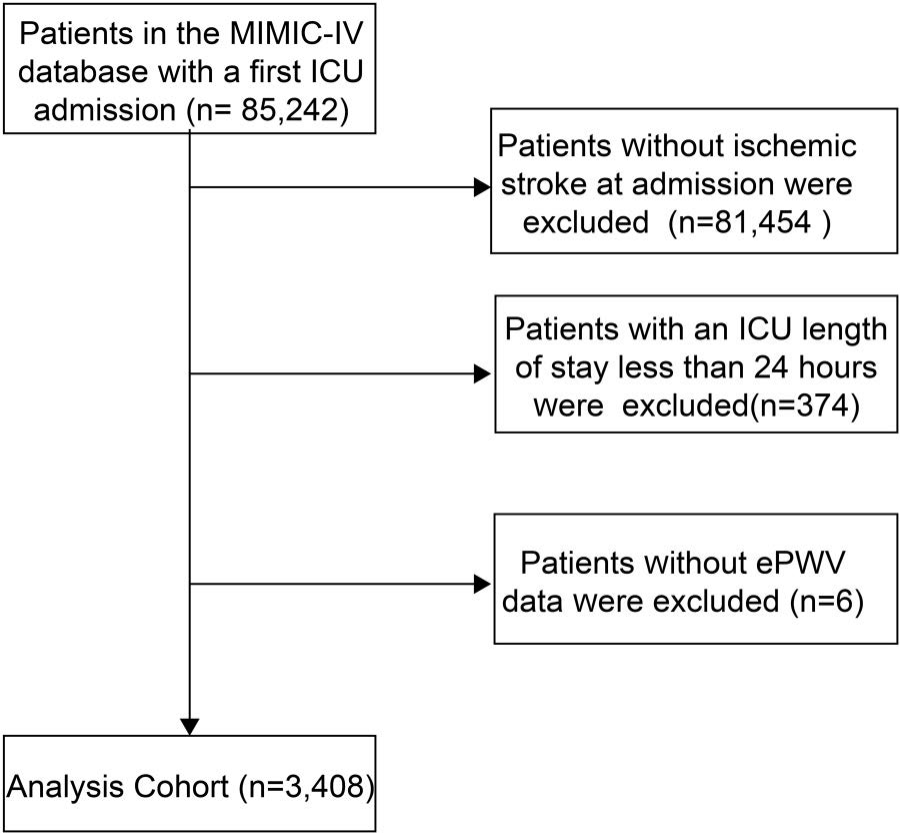
The flowchart of included patients in the study.

This study included adult patients aged 18 years and older admitted to the ICU with a diagnosis of ischemic stroke based on the International Classification of Diseases, 9th and 10th Revision [23]. Inclusion criteria required patients to have their first ICU stay recorded and a stay time in the ICU of at least 24 hours. Patients were excluded if they lacked ePWV data. Finally, 3408 patients were included and divided into three groups according to the ePWV tertiles (Fig 1).

### Measurement of ePWV and outcomes

In this study, the ePWV was calculated using the non-invasive arterial stiffness measurement equation published by the Reference Values for Arterial Stiffness Collaboration in 2010 [24]. This formula effectively captures the combined effects of age and MBP on pulse wave velocity. The MBP is determined by adding the diastolic blood pressure to 0.4 times the difference between the systolic and diastolic blood pressure. Then ePWV was derived from the formula: ePWV = 9.587 – (0.402 × age) + (4.5610 × 0.001 × age^2^) – (2.621 × 0.00001 × age^2^ × MBP) + (3.176 × 0.001 × age × MBP) – (1.832 × 0.01 × MBP) [25]. Previous studies have validated the robustness of this equation for arterial stiffness assessment by demonstrating comparable predictive value between calculated ePWV and measured cfPWV [26]. By applying this formula, we aimed to accurately assess arterial health in ischemic stroke patients, a population typically characterized by multiple cardiovascular risk factors. The primary outcomes were in-hospital and ICU 28-day mortality [27].

### Variables selection

We extracted variables as potential covariates from the database, all measured within 24 hours after ICU admission. The selection of these covariates was guided by both clinical relevance and established literature on ischemic stroke outcomes. Clinical severity scores, including SOFA, Acute Physiology Score III (APS3), SAPS II, Oxford Acute Severity of Illness Score (OASIS), and Glasgow Coma Scale (GCS), were included to comprehensively capture patient condition at admission, as these indices have been previously demonstrated to correlate with mortality risk in critically ill patients [28]. Demographic characteristics (sex, age) and comorbidities, such as heart failure (HF), chronic kidney disease (CKD), diabetes, hypertension, chronic pulmonary disease, and atrial fibrillation (AF), were selected based on their known impact on cardiovascular risk and stroke prognosis. These factors have been consistently identified in prior epidemiological studies as significant predictors of both short-term and long-term outcomes in ischemic stroke patients [29].

Laboratory parameters, including complete blood count components [red blood cell count (RBC), white blood cell count (WBC), platelet count, red cell distribution width (RDW), hemoglobin] and metabolic markers [sodium, serum creatinine, bicarbonate, potassium, chloride, total calcium], were chosen to provide a comprehensive assessment of physiological status. These markers can reflect underlying inflammatory processes, metabolic disturbances, and organ dysfunction that may influence stroke outcomes [30]. Treatment-related variables such as intravenous tissue plasminogen activator (iv-tPA), vasopressin, and mechanical ventilation were included to account for potential therapeutic interventions that might modify patient prognosis. The CCI was incorporated to adjust for the cumulative impact of pre-existing chronic conditions [31].

### Statistical analysis

Continuous variables were presented as mean ± standard deviation (SD) for normally distributed variables, median (interquartile range, IQR) for non-normally distributed continuous variables, and categorical variables were presented as numbers and percentages. Baseline characteristics were compared using t-tests or one-way analysis of variance (ANOVA) for continuous variables and Pearson’s chi-square tests for categorical variables. Missing data imputation was conducted using the missForest R package, which implements an iterative random forest algorithm for multivariate imputation [32]. This method was applied to variables exhibiting missing data rates below 30% (S1 Table), while variables exceeding this missingness threshold were excluded. The missForest algorithm is a non-parametric method that can handle non-linear relationships and complex interactions between variables. It relies on the assumption that data are missing at random (MAR), meaning that the probability of missingness depends only on observed values and not on the unobserved values themselves. In the context of this study using the MIMIC-IV database, this assumption is considered reasonable due to the database’s standardized data collection procedures. These procedures suggest that missing data are more likely due to factors in clinical practice (e.g., certain tests not being routinely performed on all patients) rather than unobserved patient-specific factors. Furthermore, variable selection was guided by clinical relevance and established literature on ischemic stroke outcomes, allowing for inference of the likelihood of missing values based on other observed variables. However, the MAR assumption is untestable and may not perfectly hold. To mitigate potential bias from this assumption, a sensitivity analysis was performed by repeating primary analyses using the original, un-imputed dataset and comparing the results.

The Kaplan-Meier curves were employed to evaluate differences in survival outcomes among tertiles of ePWV. Univariate and multivariate Cox regression models were used to examine the association between ePWV and 28-day mortality. Variables with a *P*-value < 0.05 in the univariate analysis were included in the multivariate Cox regression. Two models were constructed: Model 1 (adjusted for age, APS3, SAPS2, OASIS, SOFA, GCS, CCI), and Model 2 (fully adjusted model). Multicollinearity was assessed using the variance inflation factor (VIF), with a VIF > 10 indicating significant multicollinearity. Smooth curve fitting was also utilized to explore the association between ePWV and 28-day mortality outcomes, and threshold effect analysis was conducted to confirm the linearity of this relationship [33]. Additionally, Stratified analyses were performed across subgroups defined by age, heart failure, atrial fibrillation, mechanical ventilation, iv-tPA, and vasopressin to assess the consistency of the association. All statistical analyses were performed using R version 4.4.2 and Empowerstats (version 4.2), and a two-tailed *P*-value < 0.05 was considered statistically significant.

## Results

### Baseline characteristics

The study included 3,408 patients, with 2,927 (85.9%) surviving and 481 (14.1%) dying within 28 days of hospitalization (Table 1). Of these, 1,667 (48.91%) were female and 1,741 (51.09%) were male. Among survivors, 1423 (48.62%) were female and 1504 (51.38%) were male, while among non-survivors, 244 (50.73%) were female and 237 (49.27%) were male. There was no significant difference in sex distribution between the two groups (*P* = 0.42). Non-survivors had significantly higher ePWV values (11.19 vs. 10.57, *P* < 0.001), indicating greater arterial stiffness. They were also older (median age: 74.87 vs. 70.24 years, *P* < 0.0001) and had higher severity scores, including SOFA (5.00 vs. 3.00, *P* < 0.0001), APS3 (52.00 vs. 36.00, *P* < 0.0001), and SAPSII (43.00 vs. 32.00, *P* < 0.0001). Additionally, non-survivors exhibited a higher prevalence of heart failure (34.51% vs. 24.09%, *P* < 0.0001), CKD (24.53% vs. 18.65%, *P* < 0.01), and diabetes mellitus (38.67% vs. 32.46%, *P* < 0.01). However, no significant differences were observed in hypertension (*P* = 0.09) or chronic pulmonary disease (*P* = 0.31).

**Table 1:** Baseline Characteristics of Patients Stratified by In-Hospital 28-Day Survival Status.

Variables	Total (n = 3408)	Survive (n = 2927)	Death (n = 481)	*P*-value
ePWV	10.64 (8.86, 12.87)	10.57 (8.78, 12.76)	11.19 (9.18, 13.45)	<0.001
MBP (mmHg)	86.75 (77.88, 96.38)	87.47 (78.55, 96.83)	82.56 (75.13, 92.64)	<0.0001
Sex, n (%)				0.42
Female	1667 (48.91%)	1423 (48.62%)	244 (50.73%)	
Male	1741 (51.09%)	1504 (51.38%)	237 (49.27%)	
Age (years)	71.05 (60.06, 81.26)	70.24 (59.22, 80.59)	74.87 (64.98, 84.11)	<0.0001
Weight (kg)	77.00 (64.90, 91.63)	77.20 (65.00, 92.10)	76.00 (62.50, 89.70)	0.01
Sofa	3.00 (2.00, 5.00)	3.00 (1.00, 5.00)	5.00 (3.00, 8.00)	<0.0001
Aps3	37.00 (28.00, 51.00)	36.00 (27.00, 48.00)	52.00 (38.00, 66.00)	<0.0001
SapsII	33.00 (26.00, 42.00)	32.00 (25.00, 40.00)	43.00 (36.00, 53.00)	<0.0001
Oasis	31.00 (26.00, 37.00)	30.00 (25.00, 36.00)	37.00 (32.00, 43.00)	<0.0001
GCS	14.00 (11.00, 15.00)	14.00 (12.00, 15.00)	14.00 (9.00, 15.00)	<0.0001
CCI	7.00 (5.00, 8.00)	6.00 (4.00, 8.00)	7.00 (6.00, 9.00)	<0.0001
Heart failure, n (%)				<0.0001
No	2537 (74.44%)	2222 (75.91%)	315 (65.49%)	
Yes	871 (25.56%)	705 (24.09%)	166 (34.51%)	
CKD, n (%)				<0.01
No	2744 (80.52%)	2381 (81.35%)	363 (75.47%)	
Yes	664 (19.48%)	546 (18.65%)	118 (24.53%)	
Diabetes, n (%)				<0.01
No	2272 (66.67%)	1977 (67.54%)	295 (61.33%)	
Yes	1136 (33.33%)	950 (32.46%)	186 (38.67%)	
Hypertension, n (%)				0.09
No	1709 (50.15%)	1450 (49.54%)	259 (53.85%)	
Yes	1699 (49.85%)	1477 (50.46%)	222 (46.15%)	
Chronic pulmonary disease, n (%)				0.31
No	2822 (82.81%)	2432 (83.09%)	390 (81.08%)	
Yes	586 (17.19%)	495 (16.91%)	91 (18.92%)	
Hemoglobin (g/dL)	11.38 (10.30, 12.50)	11.40 (10.40, 12.50)	11.00 (9.50, 12.20)	<0.0001
Platelet (10^3/μL)	206.90 (168.00, 251.80)	207.00 (171.00, 252.00)	198.00 (152.00, 249.40)	0.02
RBC (10^6/μL)	3.80 (3.42, 4.21)	3.81 (3.45, 4.22)	3.69 (3.23, 4.13)	<0.0001
RDW (%)	14.10 (13.30, 15.11)	14.00 (13.28, 15.00)	14.66 (13.80, 15.90)	<0.0001
WBC (10^3/μL)	10.30 (8.20, 13.40)	10.00 (8.00, 12.80)	13.00 (10.26, 16.42)	<0.0001
Bicarbonate (mEq/L)	23.00 (21.00, 25.00)	23.00 (21.00, 25.00)	22.00 (20.00, 24.00)	<0.0001
Calcium (mg/dL)	8.62 (8.30, 9.00)	8.66 (8.30, 9.00)	8.54 (8.20, 8.90)	<0.01
Chloride (mEq/L)	106.00 (103.00, 108.00)	106.00 (103.00, 108.00)	106.00 (102.20, 110.00)	0.02
Serum_creatinine (mg/dL)	0.92 (0.80, 1.30)	0.90 (0.78, 1.20)	1.12 (0.90, 1.80)	<0.0001
Sodium (mEq/L)	140.40 (138.00, 142.40)	140.00 (138.00, 142.00)	141.00 (138.00, 144.00)	<0.0001
Potassium (mEq/L)	4.10 (3.88, 4.46)	4.10 (3.84, 4.40)	4.30 (3.92, 4.70)	<0.0001
iv-tPA, n (%)				0.19
No	2935 (86.12%)	2511 (85.79%)	424 (88.15%)	
Yes	473 (13.88%)	416 (14.21%)	57 (11.85%)	
Vasopressin, n (%)				<0.0001
No	3043 (89.29%)	2690 (91.90%)	353 (73.39%)	
Yes	365 (10.71%)	237 (8.10%)	128 (26.61%)	
Mechanical ventilation, n (%)				<0.0001
No	2096(61.50)	1957(66.86)	139(28.90)	
Yes	1312(38.50)	970(33.14)	342(71.10)	
In-hospital time (days)	8.57 (4.57, 16.30)	8.91 (4.80, 17.39)	6.31 (3.36, 11.72)	<0.0001

Missing data were imputed using the Random Forest imputation method. The following variables had missing values: Calcium (28.14%), Platelet (26.41%), RDW (26.41%), RBC (26.38%), WBC (26.38%), Hemoglobin (26.32%), Hematocrit (25.97%), Bicarbonate (22.92%), Serum creatinine (22.74%), Chloride (22.51%), Potassium (22.15%), Sodium (21.77%), and Weight (0.65%).

Abbreviations: ePWV, estimated Pulse Wave Velocity; MBP, Mean Blood Pressure; SOFA, Sequential Organ Failure Assessment; APS3, Acute Physiology Score III; SAPSII, Simplified Acute Physiology Score II; OASIS, Oxford Acute Severity of Illness Score; GCS, Glasgow Coma Scale; CCI, Charlson Comorbidity Index; CKD, Chronic Kidney Disease; RBC, Red Blood Cell; RDW, Red Cell Distribution Width; WBC, White Blood Cell; iv-tPA, intravenous tissue Plasminogen Activator.

Non-survivors demonstrated poorer laboratory profiles, including lower hemoglobin (11.00 vs. 11.40 g/dL, *P* < 0.0001), platelet counts (198.00 vs. 207.00 × 10³/μL, *P* = 0.02), and RBC counts (3.69 vs. 3.81 × 10⁶/μL, **P* *< 0.0001), alongside higher WBC counts (13.00 vs. 10.00 × 10³/μL, **P* *< 0.0001) and serum creatinine levels (1.12 vs. 0.90 mg/dL, *P* < 0.0001). Metabolic disturbances were more pronounced, with lower bicarbonate (22.00 vs. 23.00 mEq/L, **P* *< 0.0001) and calcium levels (8.54 vs. 8.66 mg/dL, *P* < 0.01), but higher potassium levels (4.30 vs. 4.10 mEq/L, *P* < 0.0001). The use of vasopressin was significantly higher in non-survivors (26.61% vs. 8.10%, *P* < 0.0001), and they had a shorter median in-hospital stay (6.31 vs. 8.91 days, *P* < 0.0001).

### Kaplan–Meier curves for In-hospital and ICU mortality

**Fig 2:**
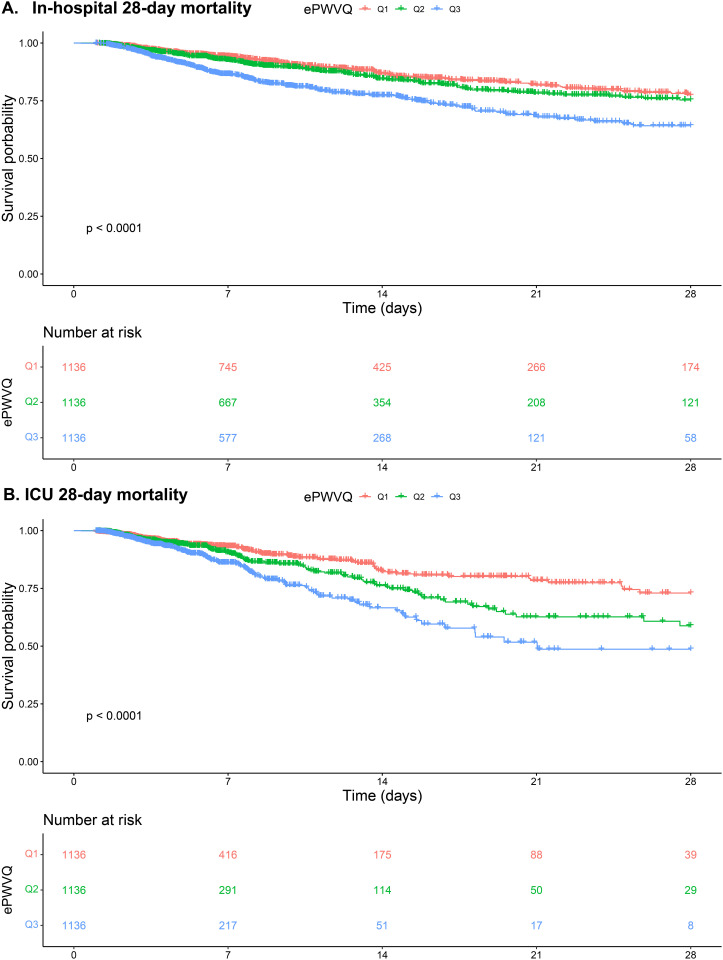
Kaplan-Meier Curves for Mortality Outcomes: (A) In-Hospital 28-day Mortality; (B) ICU 28-day Mortality.

The Kaplan-Meier curves for in-hospital and ICU 28-day mortality, stratified by ePWV categories (T1, T2, T3), demonstrate significant differences in survival probabilities (*P* < 0.0001 for both) (**Fig 2**). In both analyses, patients in the T1 group exhibited the highest survival rates, while those in the T3 group had the lowest. The survival probabilities declined steadily over the 28 days, with the T3 group showing a more pronounced decrease compared to T1 and T2. These findings indicate that higher ePWV is associated with increased mortality risk, highlighting its role as a critical prognostic indicator for both in-hospital and ICU outcomes in critically ill patients.

### Association of ePWV with In-Hospital and ICU 28-Day Mortality in Ischemic Stroke Patients

We evaluated all potential covariates using univariate Cox regression analysis (see Table 2). The results demonstrated that unadjusted ePWV was significantly associated with both in-hospital and ICU 28-day all-cause mortality (HR = 1.12, 95% CI: 1.08, 1.15, *P* < 0.0001; HR = 1.12, 95% CI: 1.07, 1.17, *P* < 0.0001, respectively). Subsequently, covariates with P < 0.05 in the univariate analysis were included in the multivariate Cox regression analysis. Table 3 presents the adjusted analysis of ePWV with in-hospital and ICU 28-day mortality among ischemic stroke patients.

**Table 2:** Univariate COX analysis of risk factors for 28-day mortality outcomes.

Variables	In-hospital 28-day mortality		ICU 28-day mortality	
	HR (95% CI)	*P*-value	HR (95% CI)	*P*-value
**ePWV**	1.12 (1.08, 1.15)	<0.0001	1.12 (1.07, 1.17)	<0.0001
**Sex**				
Male	1(Ref)		1(Ref)	
Female	1.15 (0.96, 1.38)	0.1195	1.15 (0.92, 1.43)	0.2159
**Age**	1.03 (1.02, 1.03)	<0.0001	1.02 (1.02, 1.03)	<0.0001
**APS3**	1.02 (1.02, 1.02)	<0.0001	1.02 (1.01, 1.02)	<0.0001
**SAPSII**	1.04 (1.03, 1.04)	<0.0001	1.04 (1.03, 1.04)	<0.0001
**OASIS**	1.06 (1.05, 1.07)	<0.0001	1.06 (1.05, 1.07)	<0.0001
**SOFA**	1.10 (1.07, 1.12)	<0.0001	1.10 (1.07, 1.13)	<0.0001
**GCS**	0.94 (0.91, 0.96)	<0.0001	0.94 (0.91, 0.97)	<0.0001
**CCI**	1.09 (1.06, 1.12)	<0.0001	1.09 (1.05, 1.13)	<0.0001
**Hemoglobin**	0.96 (0.92, 1.01)	0.0963	0.95 (0.90, 1.00)	0.063
**Platelet**	1.00 (1.00, 1.00)	0.1994	1.00 (1.00, 1.00)	0.5567
**RBC**	0.89 (0.77, 1.01)	0.0789	0.87 (0.74, 1.03)	0.1085
**RDW**	1.06 (1.03, 1.10)	0.0006	1.10 (1.05, 1.15)	<0.0001
**WBC**	1.01 (1.01, 1.01)	<0.0001	1.01 (1.00, 1.01)	0.0021
**Bicarbonate**	0.95 (0.92, 0.97)	<0.0001	0.95 (0.92, 0.99)	0.005
**Calcium**	0.99 (0.86, 1.13)	0.8538	1.04 (0.89, 1.22)	0.6468
**Chloride**	1.02 (1.00, 1.03)	0.0264	1.03 (1.01, 1.05)	0.0049
**Serum Creatinine**	1.06 (1.02, 1.09)	0.0012	1.07 (1.03, 1.11)	0.0013
**Sodium**	1.04 (1.02, 1.06)	<0.0001	1.05 (1.03, 1.08)	<0.0001
**Potassium**	1.43 (1.28, 1.59)	<0.0001	1.48 (1.28, 1.71)	<0.0001
**Heart Failure**				
No	1		1	
Yes	1.24 (1.03, 1.50)	0.0255	1.15 (0.90, 1.45)	0.2632
**CKD**				
No	1		1	
Yes	1.13 (0.92, 1.39)	0.2479	1.01 (0.78, 1.32)	0.9418
**Diabetes**				
No	1		1	
Yes	1.12 (0.93, 1.35)	0.219	1.06 (0.84, 1.33)	0.631
**Hypertension**				
No	1		1	
Yes	1.01 (0.84, 1.21)	0.9208	1.10 (0.88, 1.37)	0.413
**Chronic pulmonary disease**				
No	1		1	
Yes	1.04 (0.83, 1.31)	0.7236	1.08 (0.82, 1.43)	0.596
**Atrial Fibrillation**				
No	1		1	
Yes	1.45 (1.21, 1.73)	<0.0001	1.28 (1.03, 1.60)	0.0266
**Mechanical Ventilation**				
No	1		1	
Yes	2.50 (2.04, 3.05)	<0.0001	3.53 (2.56, 4.88)	<0.0001
**iv-tPA**				
No	1		1	
Yes	0.41 (0.31, 0.54)	<0.0001	0.25 (0.17, 0.37)	<0.0001
**Vasopressin**				
No	1		1	
Yes	1.87 (1.53, 2.30)	<0.0001	1.96 (1.54, 2.48)	<0.0001

Missing data were imputed using the Random Forest imputation method. The following variables had missing values: Calcium (28.14%), Platelet (26.41%), RDW (26.41%), RBC (26.38%), WBC (26.38%), Hemoglobin (26.32%), Hematocrit (25.97%), Bicarbonate (22.92%), Serum creatinine (22.74%), Chloride (22.51%), Potassium (22.15%), Sodium (21.77%), and Weight (0.65%).

Abbreviations: ePWV, estimated Pulse Wave Velocity; APS3, Acute Physiology Score III; SAPSII, Simplified Acute Physiology Score II; OASIS, Oxford Acute Severity of Illness Score; SOFA, Sequential Organ Failure Assessment; GCS, Glasgow Coma Scale; CCI, Charlson Comorbidity Index; RBC, Red Blood Cell; RDW, Red Cell Distribution Width; WBC, White Blood Cell; CKD, Chronic Kidney Disease; iv-tPA, intravenous tissue Plasminogen Activator.

**Table 3:** Multivariate Cox Regression Analysis of ePWV and Mortality Outcomes in Ischemic Stroke Patients.

Exposure	In-hospital 28-day mortality [HR (95% CI) *P*-value]		ICU 28-day mortality [HR (95% CI) *P*-value]	
	Model1	Model2	Model1	Model2
Continuous ePWV	1.14 (1.03, 1.25) 0.0086	1.16 (1.05, 1.28) 0.0033	1.25 (1.11, 1.40) 0.0002	1.31 (1.16, 1.48) <0.0001
Categorical ePWV				
T1	Ref	Ref	Ref	Ref
T2	0.94 (0.69, 1.28) 0.6826	0.98 (0.72, 1.34) 0.9166	1.64 (1.13, 2.40) 0.0097	1.60 (1.09, 2.33) 0.0156
T3	1.32 (0.86, 2.03) 0.2077	1.44 (0.93, 2.24) 0.1046	2.55 (1.50, 4.34) 0.0006	2.60 (1.51, 4.49) 0.0006
*P* for trend	0.0843	0.0432	0.0006	0.0005

Model 1: adjusted for age, APS3, SAPS2, OASIS, SOFA, GCS, and CCI;

Model 2: adjusted for age, APS3, SAPS2, OASIS, SOFA, GCS, CCI, RDW, WBC, Bicarbonate, Chloride, Serum Creatinine, Sodium, Potassium, HF, AF, Mechanical Ventilation, iv-tPA, and Vasopressin.

Missing data were imputed using the Random Forest imputation method. The following variables had missing values: Calcium (28.14%), Platelet (26.41%), RDW (26.41%), RBC (26.38%), WBC (26.38%), Hemoglobin (26.32%), Hematocrit (25.97%), Bicarbonate (22.92%), Serum creatinine (22.74%), Chloride (22.51%), Potassium (22.15%), Sodium (21.77%), and Weight (0.65%).

Abbreviations: ePWV, estimated Pulse Wave Velocity; APS3, Acute Physiology Score III; SAPS2, Simplified Acute Physiology Score II; OASIS, Oxford Acute Severity of Illness Score; SOFA, Sequential Organ Failure Assessment; GCS, Glasgow Coma Scale; CCI, Charlson Comorbidity Index; RDW, Red Cell Distribution Width; WBC, White Blood Cell; HF, Heart Failure; AF, Atrial Fibrillation; iv-tPA, intravenous tissue Plasminogen Activator.

In Model 1, after adjusting for age, APS3, SAPSII, OASIS, SOFA, GCS, and CCI, ePWV remained significantly associated with in-hospital 28-day mortality (HR = 1.14, 95% CI: 1.03, 1.25, *P* = 0.0086) and ICU 28-day mortality (HR = 1.25, 95% CI: 1.11, 1.40, *P* = 0.0002). Furthermore, in Model 2, after additional adjustments for RDW, WBC, Bicarbonate, Chloride, Serum Creatinine, Sodium, Potassium, HF, AF, Mechanical Ventilation, iv-tPA, and Vasopressin, the ePWV continued to serve as an independent predictor for in-hospital 28-day mortality (HR = 1.16, 95% CI: 1.05, 1.28, *P* = 0.0033) and ICU 28-day mortality (HR = 1.31, 95% CI: 1.16, 1.48, *P* < 0.0001).

### Analysis of ePWV: Smooth curve fitting and threshold effects on mortality outcomes.

**Fig 3:**
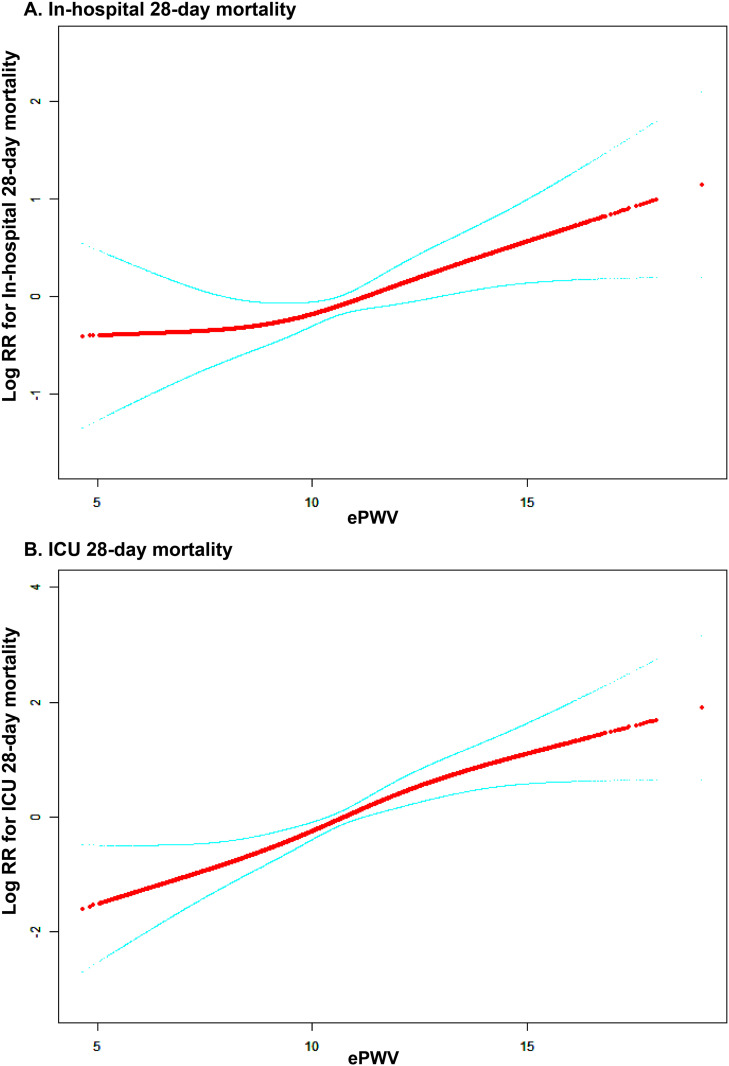
Smooth Curve Fitting of ePWV and Mortality Outcomes: (A) In-Hospital 28-day Mortality;(B) ICU 28-day Mortality. Adjusted for covariates in Model 2.

**The results of the curve fitting analysis illustrate the linear relationships between ePWV and both in-hospital and ICU mortality (**Fig 3**).** The log relative risk for mortality outcomes increases with higher ePWV values, indicating a greater mortality risk for individuals with elevated ePWV. The threshold effect analysis results in Table 4 assess the associations between ePWV and hospital 28-day mortality as well as ICU 28-day mortality using standard linear and two-piecewise linear models. The log-likelihood ratio test *P*-values for hospital mortality and ICU mortality are 0.118 and 0.202, respectively, suggesting no significant deviation from linearity in the relationships between ePWV and mortality outcomes. Overall, these findings suggest linear correlations between ePWV and both hospital 28-day mortality and ICU 28-day mortality.

**Table 4:** Threshold Effect Analysis of ePWV on In-hospital and ICU 28-day Mortality.

	In-hospital 28-day mortality	ICU 28-day mortality
**Fitting by the standard linear model**	1.16 (1.05, 1.28) 0.0033	1.31 (1.16, 1.48) <0.0001
**Fitting by the two-piecewise linear model**		
Inflection point	9.7	15.13
<Inflection point	1.04 (0.87, 1.23) 0.6980	1.34 (1.18, 1.52) <0.0001
>Inflection point	1.16 (1.05, 1.29) 0.0030	0.90 (0.48, 1.70) 0.7474
***P* for Log-likelihood ratio**	0.118	0.202

Adjusted for covariates in Model 2.

Missing data were imputed using the Random Forest imputation method. The following variables had missing values: Calcium (28.14%), Platelet (26.41%), RDW (26.41%), RBC (26.38%), WBC (26.38%), Hemoglobin (26.32%), Hematocrit (25.97%), Bicarbonate (22.92%), Serum creatinine (22.74%), Chloride (22.51%), Potassium (22.15%), Sodium (21.77%), and Weight (0.65%).

### Subgroup analyses

**Fig 4:**
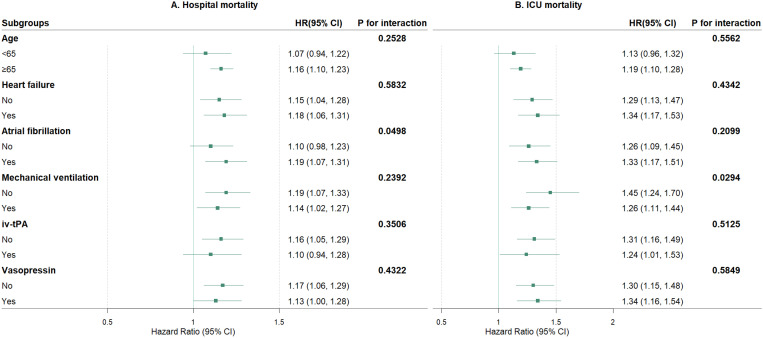
Forest Plots of Subgroup Analysis for In-hospital and ICU Mortality at 28 Days.

The subgroup analyses for hospital and ICU mortality at 28 days reveal differential impacts of clinical factors on mortality risk (Fig 4). For hospital mortality, no significant interactions were detected for age, HF, mechanical ventilation, iv-tPA, or vasopressin. However, atrial fibrillation significantly influenced hospital mortality (*P* = 0.0498), with higher risk observed in patients with AF. For ICU mortality, no significant interactions were detected for age, HF, AF, iv-tPA, or vasopressin. Notably, mechanical ventilation demonstrated a significant interaction effect (*P* = 0.0294), with higher risk observed in patients not on mechanical ventilation. These findings highlight atrial fibrillation as a significant modifier of hospital mortality and mechanical ventilation as a significant modifier of ICU mortality, while other factors did not significantly alter the relationship between ePWV and mortality outcomes.

### Sensitivity analysis

To evaluate the influence of random forest imputation on our results, we conducted a sensitivity analysis utilizing complete case analysis, which included only patients without any missing data. We subsequently re-performed the multivariate Cox regression analysis. Table 5 displays the relationship between ePWV and both in-hospital 28-day mortality and ICU 28-day mortality among patients with ischemic stroke in the complete case analysis. Consistent with the initial findings, continuous ePWV remained significantly associated with both outcomes: in-hospital 28-day mortality (Model 2: HR = 1.15, 95% CI: 1.02–1.30, *P* = 0.0194) and ICU 28-day mortality (Model 2: HR = 1.26, 95% CI: 1.10–1.46, *P* = 0.0013). Additionally, categorical ePWV continued to show significant associations with ICU 28-day mortality (T2 versus T1: *P* = 0.0335; T3 versus T1: *P* = 0.0094), with a notable trend across categories (*P* for trend = 0.0106). These findings indicate that our primary findings are relatively robust to different approaches in handling missing data.

**Table 5:** Multivariate Cox Regression Analysis of ePWV and Mortality Outcomes in Ischemic Stroke Patients (Complete Case Analysis).

Exposure	In-hospital 28-day mortality [HR (95% CI) *P*-value]		ICU 28-day mortality [HR (95% CI) *P*-value]	
	Model1	Model2	Model1	Model2
Continuous ePWV	1.14 (1.02, 1.28) 0.0201	1.15 (1.02, 1.30) 0.0194	1.25 (1.10, 1.43) 0.0010	1.26 (1.10, 1.46) 0.0013
Categorical ePWV				
T1	Ref	Ref	Ref	Ref
T2	0.98 (0.69, 1.40) 0.9127	1.04 (0.72, 1.48) 0.8480	1.63 (1.08, 2.47) 0.0198	1.57 (1.04, 2.38) 0.0335
T3	1.16 (0.70, 1.91) 0.5606	1.23 (0.74, 2.04) 0.4349	2.24 (1.24, 4.02) 0.0073	2.23 (1.22, 4.07) 0.0094
*P* for trend	0.4569	0.3799	0.0094	0.0106

Model 1: adjusted for age, APS3, SAPS2, OASIS, SOFA, GCS, and CCI;

Model 2: adjusted for age, APS3, SAPS2, OASIS, SOFA, GCS, CCI, RDW, WBC, Bicarbonate, Chloride, Serum Creatinine, Sodium, Potassium, HF, AF, Mechanical Ventilation, iv-tPA, and Vasopressin.

Abbreviations: ePWV, estimated Pulse Wave Velocity; APS3, Acute Physiology Score III; SAPS2, Simplified Acute Physiology Score II; OASIS, Oxford Acute Severity of Illness Score; SOFA, Sequential Organ Failure Assessment; GCS, Glasgow Coma Scale; CCI, Charlson Comorbidity Index; RDW, Red Cell Distribution Width; WBC, White Blood Cell; HF, Heart Failure; AF, Atrial Fibrillation; iv-tPA, intravenous tissue Plasminogen Activator.

## Discussion

The findings of this study underscore the significant association between ePWV and in-hospital and ICU 28-day mortality in ischemic stroke patients, providing novel insights into the role of arterial stiffness in critical care outcomes. The baseline demographic and clinical characteristics highlight the heterogeneity of the study population, with ePWV emerging as a key marker of vascular health. The multivariable Cox regression model confirms ePWV as an independent predictor of mortality, even after adjusting for traditional risk factors such as age, heart failure, atrial fibrillation, and mechanical ventilation. Notably, the subgroup analyses reveal that ePWV has a significant predictive value in patients with AF for hospital mortality and those not receiving timely mechanical ventilation for ICU mortality. The results highlight the potential of ePWV as a non-invasive prognostic marker in ischemic stroke.

Our findings regarding the relationship between ePWV and mortality in ischemic stroke patients align with recent evidence connecting ePWV to adverse cardiovascular outcomes. Patients with elevated ePWV face a significantly higher risk of cardiovascular mortality and major adverse cardiovascular events [34]. High ePWV levels have been linked to worse early clinical outcomes post-stroke, underscoring its importance in risk assessment for this patient group [35]. Additionally, PWV correlates with the severity of cerebral arterial damage, further underscoring its role in predicting outcomes in stroke patients [36]. While previous studies have investigated PWV as a prognostic marker, our research introduces ePWV as a non-invasive predictive measure, specifically highlighting its direct correlation with mortality risk in critically ill ischemic stroke patients. We discovered a direct correlation between ePWV and mortality risk in critically ill ischemic stroke patients, suggesting that ePWV could be a more effective prognostic tool compared to conventional risk factors.

Several mechanisms may explain the observed associations. Arterial stiffness, quantified by ePWV, disrupts cerebrovascular dynamics through complex pathophysiological mechanisms. Elevated arterial stiffness compromises blood vessels’ ability to accommodate pulsatile blood flow, resulting in heightened pulsatility within cerebral arteries [37,38]. This hemodynamic alteration substantially increases the hydraulic load on delicate microvessels, directly contributing to small vessel disease progression and escalating cerebral ischemia risk [39]. In addition to directly impacting cerebral vasculature, the elevated pulse pressure resulting from increased arterial stiffness may lead to injury in multiple organs, including the kidneys and heart. Previous studies have shown that elevated arterial stiffness reduces coronary perfusion pressure, increases left ventricular afterload, and promotes further ventricular remodeling and diastolic dysfunction [40,41]. Another investigation revealed that preoperative vascular dysfunction, characterized by increased aortic stiffness measured via PWV, is associated with a higher incidence of acute kidney injury (AKI) in patients undergoing coronary artery bypass graft surgery [42]. Mechanistically, increased arterial stiffness leads to elevated renal vascular resistance and impaired renal perfusion, both of which are critical factors in the pathogenesis of AKI [43]. Moreover, elevated ePWV may also be related to changes in inflammation and oxidative stress. Jun et al. emphasized that biochemical factors such as oxidative stress and inflammation could further exacerbate arterial stiffness, thereby impacting renal injury, especially in acute scenarios [44].

The pathological impact of arterial stiffness extends beyond hemodynamic alterations to profound structural and functional vascular changes. Higher ePWV levels are intrinsically linked to cerebral arterial calcification, serving as a robust marker of vascular health decline [45]. This calcification process not only reduces cerebral perfusion but also triggers a cascade of endothelial dysfunction and microvascular damage. The pulsatility generated by stiffened arteries initiates inflammatory responses and structural vascular remodeling, compounding the inherent risks faced by ischemic stroke patients [46]. Studies have consistently shown that elevated arterial stiffness contributes to progressive vascular damage, cognitive decline, and increased vulnerability to microvascular diseases, with particularly significant implications for patients recovering from transient ischemic attacks or strokes [47,48].

The significant interaction between ePWV and AF in-hospital mortality is particularly noteworthy. AF significantly influenced hospital mortality (*P* = 0.0498), with AF patients demonstrating a notably higher mortality risk. This association can be attributed to multiple pathophysiological mechanisms, including enhanced thrombotic potential, compromised cardiac hemodynamics, and systemic inflammatory responses [49–52]. This finding implies that patients with both elevated ePWV and AF may represent a high-risk subgroup requiring more intensive monitoring and targeted interventions. The interaction between mechanical ventilation and mortality in ischemic stroke patients reveals a critical protective mechanism of respiratory support. Providing careful ventilatory assistance can bring about significant physiological advantages, such as enhanced oxygen levels and prevention of respiratory muscle fatigue, ultimately leading to a notable decrease in mortality risk [53]. Non-ventilated patients are at a higher risk of mortality due to inadequate respiratory compensation and progressive respiratory muscle exhaustion [54]. The significant interaction effect (*P* = 0.0294) highlights the importance of early respiratory support in managing critically ill patients. These findings underscore mechanical ventilation as a pivotal life-saving intervention in severe ischemic stroke patients, serving as a critical therapeutic strategy rather than merely a supportive measure.

### Strengths and limitations

Our research has several notable strengths. Firstly, it is the first to establish a clear linear correlation between ePWV and 28-day mortality in critically ill ischemic stroke patients, suggesting a promising approach to personalized patient management. This finding complements existing literature that has linked arterial stiffness to adverse clinical outcomes, further elucidating the role of ePWV in risk stratification and management of critically ill ischemic stroke patients. The consistent association between elevated ePWV and increased mortality suggests that this non-invasive marker could serve as a valuable prognostic tool in clinical decision-making. Specifically, patients with higher ePWV values might benefit from more intensive monitoring, aggressive management of cardiovascular risk factors, and tailored interventional strategies. Secondly, our subgroup analyses revealed particularly significant predictive value in patients with atrial fibrillation and those not receiving timely mechanical ventilation, suggesting that ePWV may serve as a more refined prognostic indicator in specific high-risk patient subgroups. In patients with atrial fibrillation, where cardiovascular complications are frequent, ePWV demonstrated enhanced predictive capabilities for in-hospital mortality. Similarly, among patients who did not receive mechanical ventilation within the critical time window, ePWV emerged as a potentially crucial marker for identifying increased ICU mortality risk. These findings underscore the marker’s potential to provide more granular risk stratification beyond traditional clinical parameters, potentially guiding more personalized and targeted clinical interventions.

However, our study has several important limitations. While ePWV provides a non-invasive assessment of arterial stiffness, it may not comprehensively capture the complex pathophysiological mechanisms underlying vascular changes. Our research was conducted using the MIMIC database, a single-center cohort from an academic medical center in Boston, which potentially constrains the generalizability of our findings. The specific patient population represented in this database may not fully reflect the demographic and clinical diversity across different healthcare systems. Despite our rigorous covariate selection, the potential for unobserved confounding factors remains. The retrospective nature of the study inherently limits our ability to control for all potential confounding variables, and some clinically relevant information may not have been captured in the original medical records. Moreover, it is important to emphasize that this study is observational, and the results only indicate a correlation between ePWV and 28-day mortality, without proving causality. To further address the robustness of our findings, we performed a sensitivity analysis using complete case analysis, which included only patients with no missing data. We found that the associations between ePWV and mortality outcomes remained largely consistent with the main analysis results, suggesting that our primary findings are reasonably robust to the method used for handling missing data. However, complete case analysis also has its limitations, such as potentially reducing the statistical power of the study and introducing selection bias. Therefore, we cannot completely rule out the influence of the missing data handling method on our results.

Nevertheless, Our research provides novel insights into the relationship between arterial stiffness and outcomes in critically ill ischemic stroke patients. To address the current study’s limitations, future research should prioritize multi-center, prospective studies with more diverse patient cohorts. Validation of our findings across different healthcare settings and populations is crucial for establishing the broader clinical relevance of ePWV as a prognostic marker. Additionally, mechanistic studies exploring the complex pathways linking arterial stiffness to mortality in ischemic stroke patients would substantially contribute to our understanding.

## Conclusion

The ePWV can serve as a useful biomarker for predicting mortality risk in ischemic stroke patients, especially in patients with AF or lacking timely mechanical ventilation. These findings highlight the importance of assessing arterial stiffness in the clinical management of ischemic stroke patients. Future research should focus on validating these findings in prospective, multi-center studies, exploring the potential benefits of interventions targeting arterial stiffness, and investigating the underlying mechanisms linking ePWV to stroke outcomes. Clinically, these results suggest that ePWV assessment could be integrated into risk stratification tools to identify high-risk patients who may benefit from more intensive monitoring and targeted therapies.

## Supporting information

S1 Table Missing number of variables. (DOCX)
