# Supplementary material for: Association between Estimated Pulse Wave Velocity (ePWV) and in-hospital and ICU 28-day mortality in ischemic stroke patients: A retrospective analysis of the MIMIC-IV database
Source: PLoS One. 2025 Aug 12;20(8):e0328818. doi: 10.1371/journal.pone.0328818 (PMC12342261; doi:10.1371/journal.pone.0328818)
Supplement: S1 Table — (DOCX) [file pone.0328818.s001.docx]

**S1 Table**. Missing number of variables.

| Variables | Missing number | Percent (%) |
| --- | --- | --- |
| Calcium | 959 | 28.14 |
| Platelet | 900 | 26.41 |
| RDW | 900 | 26.41 |
| RBC | 899 | 26.38 |
| WBC | 899 | 26.38 |
| Hemoglobin | 897 | 26.32 |
| Hematocrit | 885 | 25.97 |
| Bicarbonate | 781 | 22.92 |
| Serum creatinine | 775 | 22.74 |
| Chloride | 767 | 22.51 |
| Potassium | 755 | 22.15 |
| Sodium | 742 | 21.77 |
| Weight | 22 | 0.65 |
